# A Bio-Economic Crop Yield Response (BECYR) Model for Corn and Soybeans in Ontario, Canada for 1959–2013

**DOI:** 10.1038/s41598-020-63765-3

**Published:** 2020-04-24

**Authors:** Qin Xu, Glenn Fox, Daniel W. McKenney, Gary Parkin, Zhenyi Li

**Affiliations:** 1 Department of Food, Agricultural and Resource Economics, University of Guelph, J.D. MacLachlan Building, Guelph, Ontario, N1G 2W1 Canada; 2 Canadian Forest Centre, Natural Resources Canada, 1219 Queen Street East, Sault Ste. Marie, Ontario, P6A 2E5 Canada; 3 School of Environmental Sciences, University of Guelph, 50 Stone Road East, Guelph, Ontario, N1G 2W1 Canada; 4 Department of Plant Agriculture, University of Guelph, 50 Stone Road East, Guelph, Ontario, N1G 2W1 Canada

**Keywords:** Plant sciences, Climate-change impacts

## Abstract

This paper presents estimates of the effects of changing climate on crop yields for grain corn and soybeans in Ontario, Canada, for 1959–2013. We were able to use a database that is more comprehensive with respect to explanatory variables than some previous efforts had available. Our model includes climate variables, prices, land quality, groundwater level, CO_2_ concentration, and a time trend. Our results indicate that trends in temperature and precipitation during our study period have not yet resulted in appreciable threats to crop yields in the region.

## Introduction

Climate change is a widely discussed topic, and its effect on crop production has drawn attention. The projected reduction in crop yields due to the changing climate in some regions has raised concern^1–3^. Several studies have estimated the effects of changing climate on crop yields^1,3–11^. These models include variables reflecting the effects of weather, technology and land quality. Many models, however, have not included prices, groundwater level, or CO_2_ concentration. In Table 1, we compared the variables included those previous econometric crop yield modelling research for corn and soybeans with the variables included in our study. From Table 1, we can see that we used a database that is more comprehensive with respect to explanatory variables than some previous efforts had available. We have estimated county-level yield models for grain corn and soybeans for 29 counties in southern Ontario, Canada, for the period from 1959 to 2013. Our Bio-Economic Crop Yield Response (BECYR) model includes historical weather, price, land quality, groundwater level, local CO_2_ concentration and a time trend.

**Table 1:** Comparison of Variables Included in Previous Econometric Crop Yield Modeling Research for Corn and Soybeans.

	Studied Crops, Time Periods, and Regions	Estimation Method and Functional Form	Variables Included					
			Weather	Price	Technology	CO_2_	Land Quality	Groundwater Level
This Paper	*Corn & Soybeans* 1950–2013: 29 counties in Ontario, Canada	Ordinary Least Squares (OLS) method Quadratic functional form	√	√	√	√	√ (FE/cropland area)	√ (FE/GW level)
Houck & Gallagher (1976)	*Corn* 1951–1971: US	OLS method Linear functional form	√	√	√		√ (Cropland area)	
Kaufmann & Snell (1997)	*Corn* 1969,1974,1978,1982, and 1987: counties in 8 states in the US	OLS method Quadratic functional form for weather	√		√		√ (Cropland area)	
Rickard & Fox (1999)	*Corn* 1889–1995: Ontario, Canada	OLS method Log-linear functional form Quadratic functional form	√	√	√		√ (Cropland area)	
Schlenker & Roberts (2009)	*Corn, Soybeans* 1950–2005: counties on the east of the 100° meridian in the US	Nonparametric method Step functional form for heat 8^th^ degree Polynomial for heat Piecewise linear functional form for heat Quadratic functional form for time trend	√		√		√ (FE)	√ (FE)
Cabas *et al*. (2010)	*Corn, Soybeans* 1981–2006: 8 counties in southwestern Ontario, Canada	Feasible Generalize Least Square method Just and Pope production function	√		√		√ (FE)	√ (FE)
Tolhurst & Alan (2015)	*Corn, Soybeans* 1955–2011: counties in Illinois, Indiana, and Iowa, US	Nonparametric method Quadratic functional form for precipitation	√		√			
Miao *et al*. (2016)	*Corn & Soybeans* 1977–2007: counties on the east of the 100° meridian in US	Instrumental Variable method Quadratic functional form	√	√	√		√ (FE)	√ (FE)

FE represents the county fixed effect. It is commonly used to estimate the county specific effect on crop yields, such as land quality. Groundwater level could be included in the county fixed effect, but it is not explicitly mentioned in past studies.

We use a unique weather dataset^12^ developed for this study. The weather data are from a new interpolated spatial climate dataset^12^, which was not available for previous studies. Previously, weather station data have been used in studies of this type. The limitation of weather station data is that there may be some distance between existing weather stations and the locations where crops are grown. The dataset that we have been able to access is based on a mathematical algorithm that interpolates weather station data for locations between stations. Daily temperature and precipitation are interpolated to the county-level based on the daily weather observations at 4267 weather stations across Canada^12^. We consider four definitions of the growing season^12,13^. We tested all four definitions and our modeling was based on the definition that gave us the best overall fit with our data. The growing season is characterized as starting on the day following the last occurrence of −2.2 °C at the beginning of the growing season in the spring and ending on the day preceding the first occurrence of −2.2 °C at the end of the growing season in the fall^12^. The growing season definition starts at the end of April and ends in the middle of October on average, which is consistent with the agronomic practices for corn and soybeans in studied region. For details of selecting the growing season definitions, please see the Supplementary Information.

According to our data and using the above definition, the length of the growing season in Ontario increased at an average rate of 1.45 days per decade for 1950–2013^13^. Past studies found that longer growing season contributes to higher crop yields^9,14^. So, we hypothesize that part of the increase in crop yields that has previously been attributed to advances in technology may be attributable to the increased length of the growing season.

**Figure 1:**
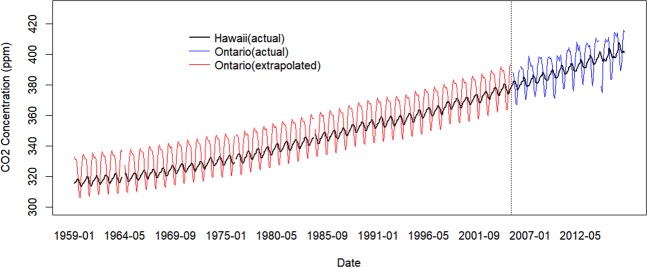
Actual and Proxy of Monthly CO_2_ Concentration (ppm) in Hawaii and Ontario for 1959–2016. Black line: the actual CO_2_ data in Mauna, Hawaii (1959–2016) from National Oceanic and Atmospheric Administration. Blue line: the actual CO_2_ data in Egbert, Ontario (2005–2016) from The World Data Center for Greenhouse Gases. Redline: the proxy of CO_2_ data in Egbert, Ontario (1959–2004). Discontinuities are due to missing values. The actual Hawaii and Ontario CO_2_ data show a similar increasing trend, but the CO_2_ concentration in Ontario varies more than in Hawaii. In addition, the CO_2_ level in Ontario reaches a peak between November and April and drops from May till July/August. The CO_2_ concentration in Hawaii peaks in May and drops from June to October. Variation in the CO_2_ concentration in Ontario reflect the reduction in photosynthesis during the winter months. Photosynthesis is less variable during the year in Hawaii. So, we regressed the monthly Ontario CO_2_ concentration on the linear corresponding value for Hawaii from 2005 to 2016. We then used the estimated coefficients from the regression to calculate the proxy serving for Ontario for 1959–2004, by substituting the Hawaii observations into the regression equation. See Table S6 for the estimated results and Fig. S2 for the residual plot in the CO_2_ regression model.

Besides, we construct a proxy variable to represent local CO_2_ concentration. There is seasonal variation of CO_2_ concentration in Ontario. The CO_2_ level reaches a peak between November and April (Fig. 1) and drops from May till July/August. Unfortunately, measurements of CO_2_ concentration in Ontario are only available for the period from 2005 to 2016. However, CO_2_ data in Mauna, Hawaii, are available back to 1958. But, the seasonality of the Hawaiian data is different from the Ontario data. The CO_2_ in Hawaii peaks in May (Fig. 1) and drops from June to October. Then, it increases slowly from October to June. And the range of CO_2_ variation in Ontario is larger than in Hawaii. The difference in seasonal variation might be due to the local climate. Hawaii is located in the tropics and the temperature is more uniform through the year compared to Ontario^15^. So, photosynthesis happens all the year round in Hawaii but is limited during the winter in Ontario.

**Figure 2:**
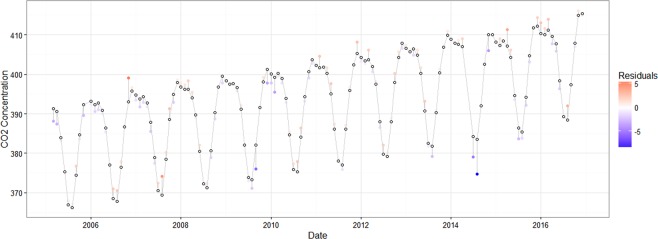
The Difference between Monthly Actual CO_2_ and Fitted CO_2_ (ppm) in Ontario from 2005 to 2016. White circles represent the fitted monthly CO_2_ in Ontario. Colored circles represent the actual monthly CO_2_ in Ontario. The distance between the white and colored circles are the residuals in the CO_2_ regression in Table S6, that is, the difference between actual CO_2_ and fitted CO_2_. The color of the colored circles represents the value of the residuals. Red means positive, and purple means negative. Larger residual has more bright color.

In the absence of Ontario data for our study period and given the lack of seasonal synchronization of the Hawaiian and Ontario data, we elected to construct a proxy of historical Ontario CO_2_ concentration for the period from 1959 to 2004. Figure 1 shows the actual CO_2_ concentration in Ontario and Hawaii as well as the proxy of CO_2_ concentration in Ontario. The proxy of CO_2_ concentration and actual CO_2_ concentration in Ontario has a similar pattern of seasonal variation. Figure 2 shows the residuals from the regression. Those residuals are generally small, so the fitted CO_2_ presents the actual CO_2_ well.

Most studies treat the effect of elevated CO_2_ on crop yields as linear^16–19^, while recent studies find a nonlinear effect of elevated CO_2_ on plant biomass^20–22^. Idso^23^ reported that the CO_2_ effect on sugar cane yield is nonlinear. But no study has found a nonlinear effect of CO_2_ on corn and soybean yields (Table 1). This is important to investigate because the prediction of future crop yields might be biased if CO_2_ effect is not correctly modeled. To compare the linear and nonlinear CO_2_ effects, we apply three versions of our models: 1) No CO_2_ effect, 2) Linear CO_2_ effect, and 3) Quadratic CO_2_ effect.

## Results and Discussion

### Crop yield response to a pest dummy variable

There was an outbreak of soybean aphids in Ontario in 2001. To measure the effect of this pest event on yields, our soybean models include a dummy variable for the year 2001. Table 2 shows the estimated effects of weather, physical, and economic variables on grain corn and soybean yields. Table 2 shows that the dummy of 2001 has a statistically significant negative effect on soybean yields as expected. Soybean yield was reduced by about 30% in 2001 due to the outbreak.

**Table 2:** Corn and Soybean Yield Models in Ontario 1959–2013 Using Fixed Effects Models with Alternative Effects of CO_2_ Concentration (a: N = 1594, b: N = 829) ***, **, and * indicate significant coefficients with significance level of 0.01, 0.05, and 0.1, respectively.

Versions of Crop Yield Model	Corn Yield Model		
	(Dependent Variable = Grain Corn Yields)		
Effects of CO_2_	No CO_2_ Effect	Linear CO_2_ Effect	Quadratic CO_2_ Effect
Independent Variables			
Precipitation Before Growing Season	−0.1091***	−0.1088***	−0.08372**
	(0.04055)	(0.04156)	(0.04095)
Precipitation During Growing Season	0.1697***	0.1697***	0.1631***
	(0.01649)	(0.01646)	(0.01645)
Degree Days During Growing Season	0.1869***	0.1867***	0.1866***
	(0.02179)	(0.02210)	(0.02160)
Corn Price Lagged One-Year	25.70***	25.71***	21.82***
	(2.057)	(2.05)	(1.958)
Fertilizer Price Index	−71.29***	−71.33***	−92.88***
	(8.952)	(8.789)	(9.069)
Trend	0.2906*	0.2563	2.737***
	(0.1691)	(0.3211)	(0.4153)
Local CO_2_ During Growing Season		0.04453	−16.35***
		(0.4454)	(1.77)
Square of Precipitation Before Growing Season	0.0002489***	0.0002483***	0.0001937**
	(0.00008557)	(0.00008762)	(0.00008565)
Square of Precipitation During Growing Season	−0.0001594***	−0.0001595***	−0.0001517***
	(0.00001669)	(0.00001668)	(0.00001733)
Square of Degree Days During Growing Season	−0.00006497***	−0.00006496***	−0.00006460***
	(0.000009012)	(0.000009010)	(0.000008757)
Square of Corn Price Lagged One-Year	−4.002***	−4.000***	−3.589***
	(0.3407)	(0.3424)	(0.3330)
Square of Fertilizer Price Index	22.59***	22.60***	28.90***
	(2.755)	(2.723)	(2.821)
Square of Trend	0.02325***	0.02275***	−0.01624**
	(0.002395)	(0.006010)	(0.007021)
Square of Local CO_2_ During Growing Season			0.02315***
			(0.002384)
Interaction of Precipitation and Degree Days	0.0002266***	0.0002265***	0.0002201***
	(0.00002119)	(0.00002121)	(0.00002066)
Adjusted R-Square	0.7869	0.7864	0.7919
F-Value	503.0	466.7	453.4
	**Soybean Yield Model**		
	**(Dependent Variable = Soybean Yields)**		
**Effects of CO**_**2**_	**No CO**_**2**_ **Effect**	**Linear CO**_**2**_ **Effect**	**Quadratic CO**_**2**_ **Effect**
**Independent Variables**			
Dummy of 2001	−11.53***	−11.36***	−11.09***
	(0.9217)	(1.000)	(0.9876)
Precipitation Before Growing Season	0.03554	0.03759	0.04349*
	(0.02401)	(0.02498)	(0.02545)
Precipitation During Growing Season	0.07197***	0.07143***	0.07058***
	(0.01073)	(0.01047)	(0.01048)
Degree Days During Growing Season	0.05434***	0.05310***	0.05020***
	(0.007541)	(0.008941)	(0.009281)
Soybean Price Lagged One-Year	7.582***	7.607***	7.405***
	(0.6085)	(0.6106)	(0.6094)
Fertilizer Price	−50.25***	−50.55***	−53.39***
	(4.79)	(4.635)	(4.623)
Trend	0.03450	−0.05598	0.6310***
	(0.07523)	(0.1765)	(0.2175)
Local CO_2_ During Growing Season		0.1208	−3.352***
		(0.2431)	(0.7144)
Square of Precipitation Before Growing Season	−0.00005486	−0.00005929	−0.00007115
	(0.00005219)	(0.00005418)	(0.0000553)
Square of Precipitation During Growing Season	−0.00005729***	−0.00005694***	−0.00005612***
	(0.000009761)	(0.000009549)	(0.000009665)
Square of Degree Days During Growing Season	−0.00001680***	−0.00001649***	−0.00001522***
	(0.000002806)	(0.000003148)	(0.000003289)
Square of Soybean Price Lagged One-Year	−0.5621***	−0.5635***	−0.5658***
	(0.05585)	(0.05606)	(0.05649)
Square of Fertilizer Price	15.24***	15.34***	16.12***
	(1.515)	(1.466)	(1.461)
Square of Trend	0.006238***	0.004846	−0.001959
	(0.001078)	(0.003289)	(0.003687)
Square of Local CO_2_ During Growing Season			0.004658***
			(0.0009189)
Interaction of Precipitation and Degree Days	0.00003381***	0.00003385***	0.00003522***
	(0.000008023)	(0.000008022)	(0.000008045)
Adjusted R-Square	0.6529	0.6522	0.6535
F-Value	124.2	115.8	109.6

The values in parentheses are standard errors. Precipitation before growing season means the precipitation in the 3 months prior to the start of the growing season. The growing season is defined as starting on the day following the last occurrence of −2.2 °C in spring and ending on the day preceding the first occurrence of −2.2 °C in fall. The degree days during the growing season refers to the degree days above 10 °C during the growing season. The crop price in Ontario and fertilizer price index in Canada is adjusted the inflation by annual all items CPI (2002 = 100). CO_2_ concentration is the combination of the proxy of CO_2_ (1959–2004) and actual CO_2_ (2005–2013) in Ontario.

### Crop yield response to technology and CO_2_

Most studies treat the effect of elevated CO_2_ on crop yields as linear^16–19^. Table 3 summarizes some of these results for corn and soybeans and compares them with our results from Table 2. For corn, we find that yield increases by 0.04% per ppm (±0.8% per ppm at 95% C.I.) of CO_2_. Past studies^17,18^ report that the changes in yields for corn range from −0.10% to 0.27% per ppm of CO_2_. For soybeans, we find that yield increases by 0.32% per ppm (±1.26% per ppm at 95% C.I.) of CO_2_. This increase is higher than previously reported results, which range from −0.00012% to 0.28% per ppm of CO_2_^16–19^. But our confidence interval covers this range. We also find that the linear CO_2_ effect on soybean is larger than that for corn. This is consistent with current crop physiology theory, which suggests that increasing CO_2_ is more beneficial for C_3_ crops than C_4_ crops^18,20^.

**Table 3:** Comparison of The Linear and Non-Linear Effects of CO_2_ on Crops in Selected Elevated CO_2_ - Plants Relationship Studies.

Studies	Time Period	Regions	Comparison of Results		
			Method	Corn (C_4_ Crop)	Soybean (C_3_ Crop; Legume)
			Linear: The percentage increase in crop yields per ppm		
Kimball (1983)	Various years	Various Countries	Review enclosure studies		0.039–0.13%
Cure & Acock (1986)	Various years	Various Countries	Review enclosure studies	−0.10–0.27%	0.062–0.11%
Long *et al*. (2006)	1992–2005 (Short-term for each crop)	Various Countries	Free-Air Concentration Enrichment Experiment (FACE)	0%	0.082%
Ziska & Bunce (2007)	Various years	Various Countries	Review enclosure and FACE studies		−0.00012–0.28%
This Paper	1959–2013	Ontario, Canada	Bio-Economic Crop Yield Response Model (BECYR)	0.04% ± 0.80%	0.32% ± 1.26%
			**Nonlinear: The direction and turning point of CO**_**2**_ **effect on plants**		
Andresen *et al*. (2017)	1998–2014	Hesse, German	Giessen Free-Air Concentration Enrichment Experiment (GiFACE)		The biomass of legumes at elevated CO_2_ (+20% above ambient) is less than that at ambient CO_2_ for 1998–2006. After 2006, the biomass at elevated CO_2_ is more than that at ambient.
Reich *et al*. (2018)	1998–2017	Minnesota, US	Free-Air Concentration Enrichment Experiment (FACE)	The biomass of C_4_ grasses was not markedly raised at elevated CO_2_ (+180ppm) in the first 12 years, but markedly raised in the subsequent 8 years.	The biomass of C_3_ grasses was markedly raised at elevated CO_2_ (+180ppm), but not in the subsequent 8 years
This Paper	1959–2013	Ontario, Canada	Bio-Economic Crop Yield Response Model (BECYR)	Corn yield decreases in the beginning. When CO_2_ reaches 353ppm (the value in 1994), yield starts to increase	Soybean yield decreases in the beginning. When CO_2_ reaches 360ppm (the value in 1998), yield starts to increase

The comparison of studied periods, regions, methods, and results are shown in each column. The top section is a comparison among studies examining the linear effect of CO_2_. The bottom section is a comparison among studies examining the nonlinear effect of CO_2_. Enclosure studies refer to the studies investigating the effect of CO_2_ in an environment which is not completely open to the field atmosphere, such as chamber and greenhouse. The Free Air Concentration Enrichment Experiment (FACE) refers to the studies to investigate the effect of CO_2_ in the field that completely open to the atmosphere. The linear CO_2_ effect in this paper is based on average Ontario crop yields for 1959–2013 (e.g. coefficient of CO_2_ concentration / Ontario corn yields 1959–2013). The 95% confidential interval is calculated. The quadratic CO_2_ effect in this paper is calculated by solving the first-order condition of crop yields in terms of the CO_2_ concentration.

Our results from a quadratic CO_2_ effect, however, tell a more complex story. We find that corn yield decreases until CO_2_ reaches 353 ppm, which was the average concentration during the growing season that occurred in 1994. Then, corn yield starts to increase at an increasing rate with CO_2_ concentration_._ Reich *et al*.^20^ also find similar nonlinear CO_2_ effect. However, our quadratic CO_2_ effect on corn yield is larger than the effect reported by Reich *et al*.^20^. The decrease of yields with higher CO_2_ could be a results of multicollinearity problem between the time trend and CO_2_ variables. Even though the nonlinear effect of CO_2_ on crop yields is found in recent studies, the nonlinear effect of CO_2_ is still a question in the field of plant physiology. Reich *et al*.^20^ indicated that the plant biomass response to elevated CO_2_ might be related to the soil N availability, but the explanation of this possible relationship is unknown yet. More researches are needed to answer this question.

For soybeans, we find that yield decreases until CO_2_ reaches 360 ppm, which is the level that occurred in Ontario in 1998. Then, soybean yield starts to increase at an increasing rate with CO_2_ concentration. Previous literature has reported inconsistent findings of a nonlinear effect of CO_2_ on soybeans^20,21^. So, we have elected to use a linear CO_2_ effect model in our results.

We acknowledge that collinearity between our proxy variable for CO_2_ and our time trend means that it is difficult to separate the individual contributions of these variables on crop yields. The correlation coefficient of these two variables is 0.99. For corn, the Variance Inflation Factor (VIF) of time trend is 19 and the VIF of CO_2_ is 33. For soybeans, the VIF of time trend is 18 and the VIF of CO_2_ is 33. All the above values indicate that there is a multicollinearity problem between time trend and CO_2_ variables. So, we cannot compare the individual contributions of our proxy variable for CO_2_ and the time trend on crop yields.

### Crop yield response to weather variables

Our results (Table 2) indicate that corn and soybean yields respond to the precipitation before the growing season in different ways. Corn yield decreases with more precipitation before the growing season while soybean yield increases. Precipitation before the growing season can impair the timeliness of tillage operations and reduce soil temperature^24^. This can delay corn planting and germination^24^. Soybeans can be planted later than corn in Ontario without incurring a yield penalty. So excess moisture in the spring can shift planted area away from corn and toward soybeans and it can reduce yields in the land that is planted with corn. In average, the increasing precipitation before the growing season reduces 0.00045% of corn yield per year and increases 0.0017% of soybean yield per year.

A longer growing season leads to an increase in precipitation during the growing season and also an increase in degree days during the growing season because there are more days for which precipitation and temperature are measured. Degree days during the growing season is the accumulation of the positive difference between daily average temperature and base temperature (10 °C) during the growing season. According to the plant physiology theory, when plants received either insufficient or excess moisture and heat, the physiology processes of plant would be impeded^24–26^. So, when crops receive insufficient moisture or heat, we expect crop yields increase as more precipitation and degree days during the growing season. But, when crops receive excessive moisture and heat, we expect crop yields decrease as more precipitation and degree days during the growing season. We found that corn and soybean yields increased at a decreasing rate as the precipitation and degree days during the growing season increased (Table 2). Given the quadratic functional form in our model, these results imply that there is a level of growing season precipitation and growing season degree days at which yields reach a maximum with respect to each variable. We call these maxima peak yields. Peak yields with respect to water and heat vary across years due to interaction effects (Table 2). To explore if recent precipitation and degree days had exceeded the water and heat levels associated with peak yields, we elected to study the time period from 2009–2013.

For our linear CO_2_ effect model, the peak yield with respect to precipitation is reached when the precipitation during the growing season reaches 599 mm per season for corn and 655 mm per season for soybeans. The peak yield with respect to degree days is achieved when degree days during the growing season reaches 1507 °C days for corn and 1651 °C days for soybeans. The average precipitation and degree days during the growing season for 2009–2013 were 491 mm and 1269 °C respectively. For the most recent 5 years in our data set, it appears that climate has not yet resulted in appreciable threats to yields of corn and soybeans in Ontario. In average, the increasing precipitation and degree days during the growing season increase 0.084% per year in corn yield per year and increase 0.13% per year in soybean yield (Table 4). Climate projections for Ontario suggest a wetter and warmer growing season along with significant changes in return rates for extreme events for 2020–2070^12,27–29^. More frequent extreme events could be an important driver of future yields. Our ongoing research is investigating this possibility through spatial stochastic simulation modeling.

**Table 4:** The Change in Crop Yields Attributable to Each Time-Variant Variable per Year in Average as A Share of the Grain Corn and Soybean Yields in 1959.

Crops	Grain Corn		Soybeans	
Effects of CO_2_	No CO_2_ Effect	Linear CO_2_ Effect	No CO_2_ Effect	Linear CO_2_ Effect
Variables				
Precipitation before growing season	−0.00045%	−0.00045%	0.0017%	0.0017%
Precipitation and degree days during the growing season	0.085%	0.084%	0.13%	0.13%
Crop price lagged one-year	−0.22%	−0.22%	−0.33%	−0.33%
Fertilizer price index	0.022%	0.022%	0.065%	0.065%
Time trend	1.1%	1.0%	0.77%	0.43%
CO_2_ concentration		0.043%		0.34%

The percentage of crop yields in 1959 results from each variable is calculated as the change in crop yield attributable to one variable divided by the fitted crop yield in 1959. We combine the effects of precipitation and degree days during the growing season because of the interaction effect between precipitation and degree days.

The literature on the effects of climate change on crop production in Ontario is inconclusive. Brklacich and Smith^30^ reported that increased future variation in climate might reduce crop production in Southern Ontario. However, Weber and Hauer^31^ argued that Ontario agriculture would benefit from projected changes in climate. Cabas *et al*.^9^ found that increased variation in temperature and precipitation might decrease crop yields in Ontario for 1981–2006. But they indicated that the longer growing season might offset the negative effect and result in higher crop yields. Our results suggest that projected changes in climate do not currently pose a threat to corn and soybean yields in Ontario. In the United States, Schlenker and Roberts^1^ found that climate change threatens corn, soybean and cotton yields for 1950–2005. Miao *et al*.^3^ found that climate change negatively affects corn and soybean production in the United States for 1977–2007. They also indicated that the omission of economic variables might have led to the overestimation of the effect of climate change on corn and soybean yields. Recently, Butler *et al*.^14^ found that the longer growing season and decreased peak temperatures during the growing season have been beneficial for corn yields in the U.S since 1981.

### Crop yield response to economic variables

Agricultural economists have argued that crop yields are influenced by output and input prices^8^. However, price variables have been omitted from several recent studies on the effects of climates on crop yields (Table 1). The effect of the crop price on yields is theoretically ambiguous. Higher expected crop prices might induce farmers to use purchased inputs like fertilizer more intensively, increasing yields. But a higher expected crop price might also induce farmers to increase area planted to that crop, expanding production onto less suitable soils, reducing yields. A higher fertilizer price would result in less intensive fertilizer use and lead to a reduction in yields^3,8^. However, increased fertilizer prices might cause farmers to shift planted area having lower corn yield away from corn and into soybeans^3^. Thus, the remaining land with high corn yield might increase the average corn yield^3^. Therefore, the effect of fertilizer price on yields is theoretically ambiguous.

As corn and soybeans are planted in the spring, farmers do not know the crop prices that they will receive until after they plant. We used the crop price lagged one-year in our models to represent farmers’ expectations. We find that corn and soybean yields increase at a decreasing rate as crop prices increase (Table 2). We also find that decreasing of crop price lagged one-year decreases 0.22% per year in corn yield and decreases 0.33% per year in soybean yield (Table 4).

We used the current year index of fertilizer price as the input price in our models. We find both positive and negative effects of fertilizer price on crop yields for our study period. We find that when the fertilizer price index is lower than approximately 160 (100 = dollars in year 2002), corn and soybean yields decrease at a decreasing rate as the fertilizer price goes up (Table 2). When the fertilizer price index is higher than approximately 160 (100 = dollars in year 2002), crop yields increase at an increasing rate as the fertilizer price gets higher (Table 2). In average, decreasing fertilizer price increases 0.022% per year in corn yield and 0.065% per year in soybean yield (Table 4).

## Conclusion

This paper builds on previous research on the effects of climate on corn and soybean yields in Ontario, Canada. We constructed a database that is more comprehensive with respect to explanatory variables included in the estimation. Our model includes climate variables, prices, land quality, groundwater level, CO_2_ concentration, and a time trend. We use a unique annual panel dataset for 29 counties in Ontario for the time period from 1959 to 2013. Our results indicate that historical trends in temperature and precipitation have not yet resulted in appreciable threats to crop production in Ontario, Canada. However, the prospect of more frequent extreme climate events calls for further research on the effect of climate on crop yields. We are undertaking spatial stochastic simulation modeling to investigate this possibility.

A growing body of evidence suggests that changes in climate are or will constitute a threat to crop production in some major crop producing regions of the world^11,32,33^, including the United States^1–3^. But the effect of changing climate on crop production is regional specific. Our research as well as previous work at the national level in Canada^31^ suggest that historical trends in temperatures and precipitation have not yet had an adverse effect on crop yields in Canada. Weber and Hauer^31^ found that all provinces in Canada would benefit from climate change. The above discussion suggests that for a temperate climate country like Canada, threats to crop production from climate have yet to materialize. This suggests the possibility that climate is causing changes in regional or national comparative advantage in agriculture. This may have important implications for future agricultural trade and global food security.

Past studies^11,34,35^ use a fixed fertilization effect of CO_2_ from experimental studies to predict future crop production. However, the fertilization effect of CO_2_ may change by time period, crops, and studied locations. Long-term CO_2_-plant experimental data are not easy to obtain. Our BECYR model constructs a synthetic CO_2_ proxy. This method contributes the estimation of crop yield model when actual long-term CO_2_ data are unavailable.

## Method and Data

We model crop yield as a function of precipitation and degree days during the growing season, precipitation before the growing season, cropland quality, groundwater level, crop price, fertilizer price, local CO_2_ concentration and a time trend. Grain corn and soybeans are the two most valuable field crops in Ontario. Our panel data extend from 1959 to 2013 for 29 counties in Ontario, Canada. A quadratic functional form is used for every variable except the dummy variables to test for non-linear effects. We estimated two types of model: a pooled regression model and a fixed county effects model. The time-invariant variables, cropland quality and groundwater, are included in the pooled regression model but not in the fixed county effects model. The advantage of a Fixed Effects model is that it to capture the effect of county-level variation in local factors, such as soil type, groundwater, topography and others. Due to poor data quality for these local factors, the pooling regression models are less likely to capture their effects. In addition, the F-test of fixed effect model is higher than that of pooling regression model. So, our study focusses on the results from county level fixed effect models.

The Fixed Effects model allows the intercept term to vary across the 29 counties. Note that, the time-specific effect is not discussed here since we focus on the specific effects across counties and we have included the variable of time trend. This model assumes that county-specific effect is unique for each county but constant over time. Allowing the intercept to vary across counties is useful to capture the effects of other factors not included in each county. So, parameters ${\alpha }_{c}$ vary across counties but not over time, and parameters ${\beta }_{i}$, ${\gamma }_{i}$ and $\theta$ are constant for all counties and years. The model also includes an individual error term ${e}_{t,c}$, which varies across both counties and times. Equation (1) shows the Fixed Effects model for corn yield with quadratic CO_2_ effect:

1 $$\begin{array}{rcl}{Y}_{t,c} & = & {\alpha }_{1}{D}_{1}+{\alpha }_{2}{D}_{2}+\cdots +{\alpha }_{29}{D}_{29}+{\beta }_{1}PBG{S}_{t,c}+{\beta }_{2}PREC{I}_{t,c}+{\beta }_{3}D{D}_{t,c}+{\beta }_{4}{P}_{t-1}+{\beta }_{5}P{F}_{t}\\  &  & +\,{\beta }_{6}{T}_{t}+{\beta }_{7}C{O}_{2t}+{\gamma }_{1}PBG{{S}_{t,c}}^{2}+{\gamma }_{2}PREC{{I}_{t,c}}^{2}+{\gamma }_{3}D{{D}_{t,c}}^{2}+{\gamma }_{4}{{P}_{t-1}}^{2}\\  &  & +\,{\gamma }_{5}P{F}_{t}^{2}+{\gamma }_{6}{T}_{t}^{2}+{\gamma }_{7}C{{O}_{2t}}^{2}+\theta (PREC{I}_{t,c}-\overline{PRECI})(D{D}_{t,c}-\overline{DD})+{e}_{t,c}\end{array}$$

where

${Y}_{t,c}$ is the annual grain corn yield in year *t* and county *c*;

${D}_{c}$ (*c* = 1, 2, …, 29) is the dummy variable for each of the 29 counties;

$PBG{S}_{t,c}$ is the precipitation before the growing season, which in this context is the 3 months prior to growing season in year *t* and county *c*;

$PREC{I}_{t,c}$ is the total precipitation during the growing season in year *t* and county *c*;

$D{D}_{t,c}$ is the degree days during the growing season with 10 °C base temperature in year *t* and county *c*;

${P}_{t-1}$ is the corn price in Ontario for the previous year *t-1*;

$P{F}_{t}$ is the fertilizer price index in Canada for the current year *t*;

${T}_{t}$ is the time trend;

$C{O}_{2t}$ is the proxy of CO_2_ concentration in Ontario for the year t;

$\overline{PRECI}$ is the mean of $PREC{I}_{t,c}$ for all studied counties and years;

$\overline{DD}$ is the mean of $D{D}_{t,c}$ for all studied counties and years.

The model considers the linear and quadratic effect of each factor as well as the centering interaction between precipitation and degree days. The centering interaction is used to reduce the correlation between the variables and their interaction term. The estimated results are robust for heteroskedasticity and autocorrelation.

For validation, we did hold-out validation and out-of-sample simulation. The results from both methods indicate that our crop yield models for grain corn and soybeans are reliable. For details of the validation, please see the Supplementary Information.

Many counties in Ontario experienced changes in county boundaries from 1959 to 2013. There is a concern that yield data at the county level significantly change due to the historical county boundary changes. Xu^28^ had determined the effect of county boundary changes on yield data and adjusted county boundaries. We used the adjusted county boundaries from her work for our crop yields and climate data.

Data on corn yields (Bushels/Acre) and soybean yields (Bushels/Acre) were collected from 1959 to 2013. The yield data were obtained from Agricultural Statistics for Ontario. Due to county boundary changes described above, we did retroactive adjustment to the original crop yields data for the years prior to the boundary changes for each county. For the counties with county boundary adjustment, the harvest area weighted average crop yields data are calculated for the new county boundary. Equation (2) shows the formula of the harvest area weighted average crop yields:

2 $$Yiel{d}_{New}=Yiel{d}_{1}\times \frac{Are{a}_{1}}{Are{a}_{1}+Are{a}_{2}}+Yiel{d}_{2}\times \frac{Are{a}_{2}}{Are{a}_{1}+Are{a}_{2}}$$

where

$Yiel{d}_{New}$ is the harvest area weighted average crop yields in the new combined county;

$Yiel{d}_{k}\,(k=1,2,\ldots )$ is the crop yield in county k;

$Are{a}_{k}$ is the crop harvest area in county k.

For soybeans, yield data are not available for all counties over the whole study period. From the 1950s to 1970s, the planting of soybeans was restricted to southern Ontario due to that region having the longest and warmest growing season in Canada^36^. Advanced breeding technology allowed farmers to grow soybeans outside of Southern Ontario starting in the 1980s^36^. For this reason, very few soybeans were planted outside of Southern Ontario prior to that period. As a result, the data for soybean production and harvest area is very small in the early period and missing in some years. There is a concern that these data for small-scale soybeans production cannot well represent the soybean production in Ontario and therefore widens the variance of soybean yields. Therefore, we excluded soybean yields data for counties with harvest area less than 1,500 acres per year. In addition, to keep the continuity of soybean yields data, we excluded the soybean yields data in the early period until no data were missing. So, the Soybean yield data are missing in some years in some counties until the year of 1996. We estimated soybean yield model for two different periods: 1) the period of 1959–2013, which is unbalanced panel; and 2) the period of 1996–2013, which is balanced panel. The results from these two tests are similar. The estimated coefficients show the same sign and level of significance. Since the results are similar, we used the model estimated the period of 1959–2013, as it is based on more observations which allow us to better measure the effect of changing climate on crop yields over time.

Climate data were obtained from Natural Resources Canada (NRCan)^12,27^. The data includes historical precipitation before the growing season (mm) (*PBGS*), precipitation during the growing season (mm) (*PRECI*) and degree days during the growing season (°C) (*DD*) for the period.

Economic factors include real crop prices and fertilizer price index. We use the Consumer Price Index (CPI) from Statistics Canada, CANSIM Table 326-0021 to convert nominal crop and fertilizer prices to real prices to adjust inflation. As corn and soybeans are planted in the spring, farmers do not know the crop prices that they will receive until after they plant. So, the 1-year lag of crop price is used as the expected crop price in the models for corn and soybeans. Corn price data (dollars per metric ton) in Ontario from 1949 to 2012 were obtained from Statistics Canada CANSIM Table 001-0010 and Table 002-0043 for 1949 to 1984 and 1985 to 2012, respectively. Soybean price data (dollars per metric ton) in Ontario from 1949–2012 were obtained from Statistics Canada CANSIM Table 001-0010, Publication 22-007 and CANSIM Table 002-0043, for 1949 to 1984, 1985 to 1991, 1992 to 2012, respectively. The monthly price data are averaged to obtain annual data.

The composite fertilizer price index in Canada is used as the nominal fertilizer price ($P{F}_{t}$). The fertilizer price index (index 1998=100) in Canada from 1959 to 2013 was obtained from Statistics Canada Publication 62-004, CANSIM Table 328-0001, CANSIM Table 328-0014 and CANSIM Table 328-0015, for 1959 to 1961, 1961 to 1998, 1998 to 2002, and 2002 to 2013. The quarterly fertilizer price index is averaged to obtain annual observations.

Technology development plays an important role in crop production, such as the development in plant breeding, and management practices in production systems. A time trend is used as a proxy of technology in this study for each crop. The time trend is a number sequence from 1 to 55.

The monthly CO_2_ data in Egbert, Ontario for 2005–2016 is collected from The World Data Center for Greenhouse Gases (WDCGG). The monthly CO_2_ data in Mauna, Hawaii is collected from the National Oceanic and Atmospheric Administration (NOAA). We used the following procedure to construct a proxy of historical Ontario CO_2_ concentration for the period from 1959 to 2004. We first calculated the correlation coefficients for monthly average CO_2_ concentration in Ontario and Hawaii for 2005–2016. The 12 correlation coefficients are all above 0.85. Then, we regressed the monthly Ontario CO_2_ concentration on monthly Hawaii CO_2_ concentration for 2005–2016. We used a linear monthly fixed effects model. We found that the adjusted R-square is 0.97, which shows a high goodness of fit. In addition, the estimated coefficients for Hawaii CO_2_ concentration and monthly fixed effects are all statistically significant. We then used the estimated coefficients to construct a proxy for Ontario CO_2_ concentration for 1959–2004. For the details of the methods and estimation results, see the Support Information (Tables S5–S6).

## Supplementary information

Supplementary Information.

## Data Availability

The data and R code used in this paper are available at the University of Guelph data depository system (10.5683/SP2/6OJSHE). All the other method and materials are present in the paper or supplementary information.
