# Supplementary material for: A Bio-Economic Crop Yield Response (BECYR) Model for Corn and Soybeans in Ontario, Canada for 1959–2013
Source: Sci Rep. 2020 Apr 24;10:7006. doi: 10.1038/s41598-020-63765-3 (PMC7181836; doi:10.1038/s41598-020-63765-3)
Supplement: Supplementary file 1 — Supplementary Information. [file 41598_2020_63765_MOESM1_ESM.pdf]

Supplementary Information for

A Bio-Economic Crop Yield Response (BECYR) Model for Corn and Soybeans in Ontario, Canada for 1959-2013

Qin Xu*, Glenn Fox, Dan McKenney, Gary Parkin, Zhenyi Li

* Corresponding author. E-mail address: xuq@uoguelph.ca

This file includes:

The Selection of Growing Season Definitions
Out-of-Sample Simulation
Hold-Out Validation
Table S1 to S6
Figure S1 to S4

The Selection of Growing Season Definitions

We considered four definitions of the growing season. Table S1 defines and compares the four definitions of the growing season. These four growing season definitions have been commonly used in the literature. Each definition is based on specific temperature thresholds. We estimated our models with each of the four definitions and chose the definition that gave us the best fit to our data, which is the one named "Medium Growing Season" in the Table S1. For both grain corn and soybeans, the estimated results do not vary substantially across the four growing season definitions. The sign and statistical significance level of most estimated coefficients are constant across four growing season definitions. Therefore, we use two criteria to determine the most suitable growing season definitions: the general agronomic practice of farmers growing corn and soybeans in Ontario and the value of adjusted R-squared among four definitions.

We selected the "medium growing season" definition for two reasons. First, the values of adjusted R-squares across four definitions are close but that of the "medium growing season" fits slightly better than the others. Second, the "medium growing season" definition is consistent with typical agronomic practices for corn and soybeans in the studied region. Table S2 shows the average start date and average end date of each growing season definition for our studied time period. Table S2 shows that the growing season definition we chose starts at the end of April and ends at the middle of October on average. Based on our communication with local farmers, local agronomists, and the field crop guideline by Ontario Ministry of Agriculture, Food and Rural Affairs (2017)[1], the actual planting date of corn and soybeans ranges between late April and the middle of May. When the spring conditions are favorable, late April or early May is preferred

due to the yield advantage. The actual harvesting date of corn and soybeans generally ranges between middle of October and middle of November, depending on the weather conditions.

The degrees of freedom are not affected by the selection of growing season definition, since the number of observations and the number or parameters to be estimated are the same in the four versions of each yield model. There are no coefficients that are estimated that are specific to the specification of the length of the growing season.

Out-of-Sample Simulation

We did an out-of-sample simulation for 2014 – 2016 and compared the simulation yields with the actual yields. But, since we only have interpolated spatial weather data till 2013. The weather data for 2014 - 2016 simulation data, which cannot reflect the actual weather condition. So, the weather data for 2014 - 2016 have lower quality than the weather data for 1959-2013. For the time period of 2014-2016, we extrapolated the county-level historical linear trend of weather data and set up the mean and standard deviation of each weather variable in each county for each year. The price variables were assumed constant since no one knows what the trend of prices are in future. We used Monte-Carlo method. We run 500 simulations for each crop, each county, and each year. Through the above steps, we obtained the distribution of out-of-sample simulation corn and soybean yields for 2014-2016 for 29 counties in Ontario, Canada.

Figure S1 shows the comparison of the provincial means of actual and simulation corn and soybean yields in Ontario for 2014-2016. The simulation yields are around the actual yields, which do not either underestimate or overestimate the actual yields.

Figure S2 shows the comparison of the county-level means of actual and simulated corn yields in Ontario for 2014-2016. In each year, the simulation yields are close to the actual yields. For 2016, the differences between actual and simulation yields were larger in some counties, especially in the Central and Eastern Ontario. This might be because the weather in Ontario in 2016 was much drier than average years, which resulted in lower actual yields. Recall the limitation of weather data quality for 2014 -2016 we mentioned above, if we had the actual weather data for 2016 rather than simulation data, the difference between actual and simulation yields might be less.

Figure S3 shows the comparison of the county-level means of actual and simulated soybean yields in Ontario for 2014 – 2016. The results for soybeans are similar to those for corn. In each year, the simulation yields are close to the actual yields, do not appear to over or underestimate actual yields. For 2016, the differences between actual and simulation yields are large in some counties. This might because of the limitation of weather data quality for 2014 – 2016, which we mentioned above.

Figure S4 shows the average deviation of simulation yields as percentage of actual yields for corn and soybeans in the time period of 2014-2016 across 29 counties. In the figure, the mean percentage deviation for each crop and each year is close to 0, which means that our crop yield models were consistent with actual yields. We think that the percentage deviations for 2016 are due to data quality problems.

Table S3 shows the comparison of the Mean Squared Error (MSE) between the studied period and the out-of-sample simulation period for corn and soybean yields. For both crops, the

MSEs for 1959-2013 are less than the MSEs for 2014-2016. Recall the limitation of weather data quality for 2014 -2016 we mentioned above, if we had the actual weather data for 2016 rather than simulation data, the MSEs for 2014-2016 might be lower.

Hold-Out Validation

We used the hold-out validation method to validate the crop yield models for grain corn and soybeans. We randomly split the 55-year data for 1959-2013 into testing data and training data. The standard practice in the literature is to use 20%-40% of an entire data set as testing data in validation. We randomly selected 17 years of data as testing data, which accounts for 31% of the entire 55-year time span of our study. The remaining 38 years of data are our training data. In both the testing data and the training data we used observations from all 29 counties in order to maintain our county-specific fixed effect estimation approach. In the validation, for each crop, we first estimated the yield models with our training data. We then calculated the differences between predicted yields from the estimation and actual crop yields from the training data (i.e. residuals). Second, we calculated the Mean Squared Error (MSE) for the training data by summing up the square of these differences and divided by the number of observations in the training data. Third, we calculated the Root Mean Squared Error (RMSE) for the training data by calculating the square root of the MSE for the training data. Fourth, we used the estimated coefficients for the training data with the testing data to calculate predicted yields. We then calculated the differences between these calculated yields and observed yields for each year for each county in the testing data. Fifth, we calculated the Mean Squared Error (MSE) for testing

data by summing up the square of these differences and divided by the number of observations in the testing data. Finally, we calculated the Root Mean Squared Error (RMSE) for the testing data by calculating the square root of the MSE for the testing data. For corn, we found that the RMSE for the training data and the testing data are 12.82 bushels/acre and 13.54 bushels/acre, respectively. Since the normalized RMSE (NRMSE) here is defined as the RMSE divided by the mean of crop yields, the NRMSE for the training data and the testing data are 13.6% and 13.3%, respectively. For soybeans, we found that the RMSE for the training data and the testing data are 4.18 bushels/acre and 4.23 bushels/acre, respectively. So, the NRMSE for the training data and the testing data are 11.7% and 12.1%, respectively. From the results in Table S4, the NRMSEs for different datasets are in the range of 11.2%-13.6%. There is no upper limit of NRMSE, but a lower value would be better. Liu et al (2013)[2] considered a NRMSE value less than 15% as "good," a value between 15% and 30% as "moderate" and a value greater than 30% as "poor." Our calculated NRMSEs for alternative data are all less than 15% and fall in the "good" range specified by Liu et al (2013)[2]. We conclude, on this basis, that our models are validated.

References

1. Ontario Ministry of Agriculture, F. and R. A. *Agronomy guide for field crops - publication 811*. (2017).
2. Liu, S. *et al.* Modelling crop yield, soil water content and soil temperature for a soybean-maize rotation under conventional and conservation tillage systems in Northeast China. *Agric. Water Manag.* **123**, 32–44 (2013).
3. Pedlar, J. H. *et al.* A Comparison of Two Approaches for Generating Spatial Models of Growing-Season Variables for Canada. *J. Appl. Meteorol. Climatol.* **54**, 506–518 (2015).

Table S1: The Definitions of Four Alternative Growing Seasons

Name of Growing Season Definition	Definition
5 Consecutive Day Average Temperature Growing Season	Starts when the mean daily temperature is greater than or equal to 5°C for 5 consecutive days beginning March 1, and ending when the one-day average minimum temperature is less than -2°C beginning August 1.
Short Growing Season	Starts on the following day of last occurrence of 0°C in spring and ends on the preceding day of the first occurrence of 0°C in fall
Medium Growing Season	Starts on the following day of last occurrence of -2.2°C in spring and ends on the preceding day of the first occurrence of -2.2°C in fall
Long Growing Season	Starts on the following day of last occurrence of -4.4°C in spring and ends on the preceding day of the first occurrence of -4.4°C in fall

Source: Pedlar et al (2015)[3]

Table S2: The Average Start Date and End Date of Four Alternative Growing Season Definitions

Name of Growing Season Definition	Average Start Date	Average End Date
5 Consecutive Day Average Temperature Growing Season	Mid of April	Mid of November
Short Growing Season	Mid of May	Early of October
Medium Growing Season	End of April	Mid of October
Long Growing Season	Mid of April	Early of November

Source: Authors' calculation based on data from Pedlar et al (2015)[3].

Figure S1: Provincial Level Comparison Means of Actual Yields and Simulation Yields for 2014-2016

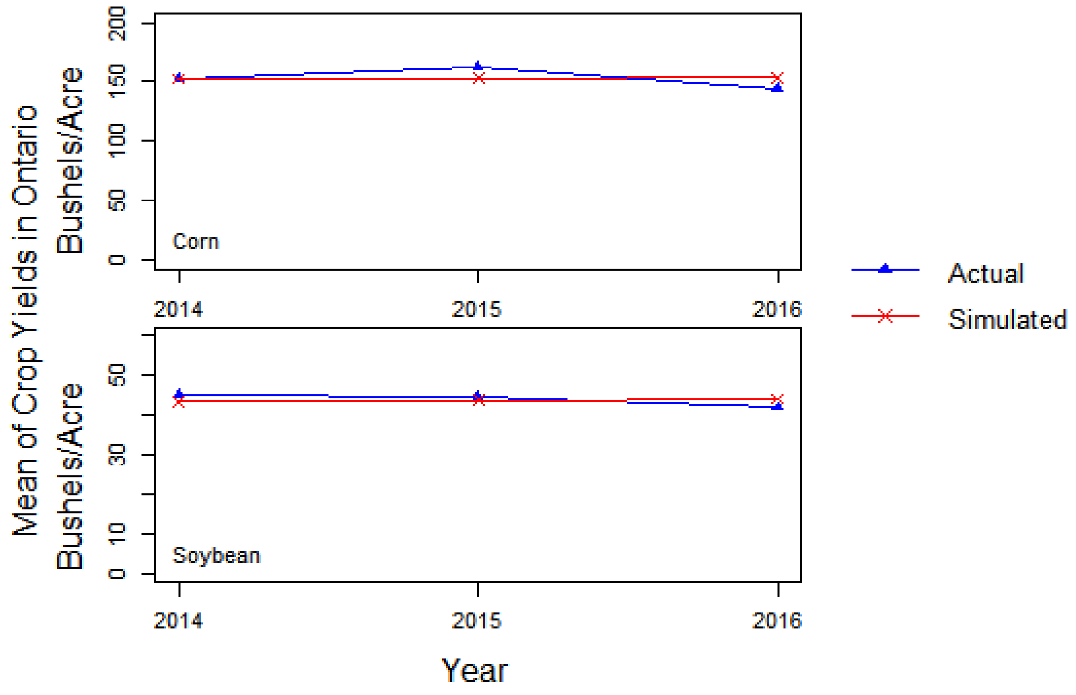

Source: Authors’ calculation

Figure S2: County-Level Comparison Means of Actual Yields and Simulation Yields for Corn for 2014-2016

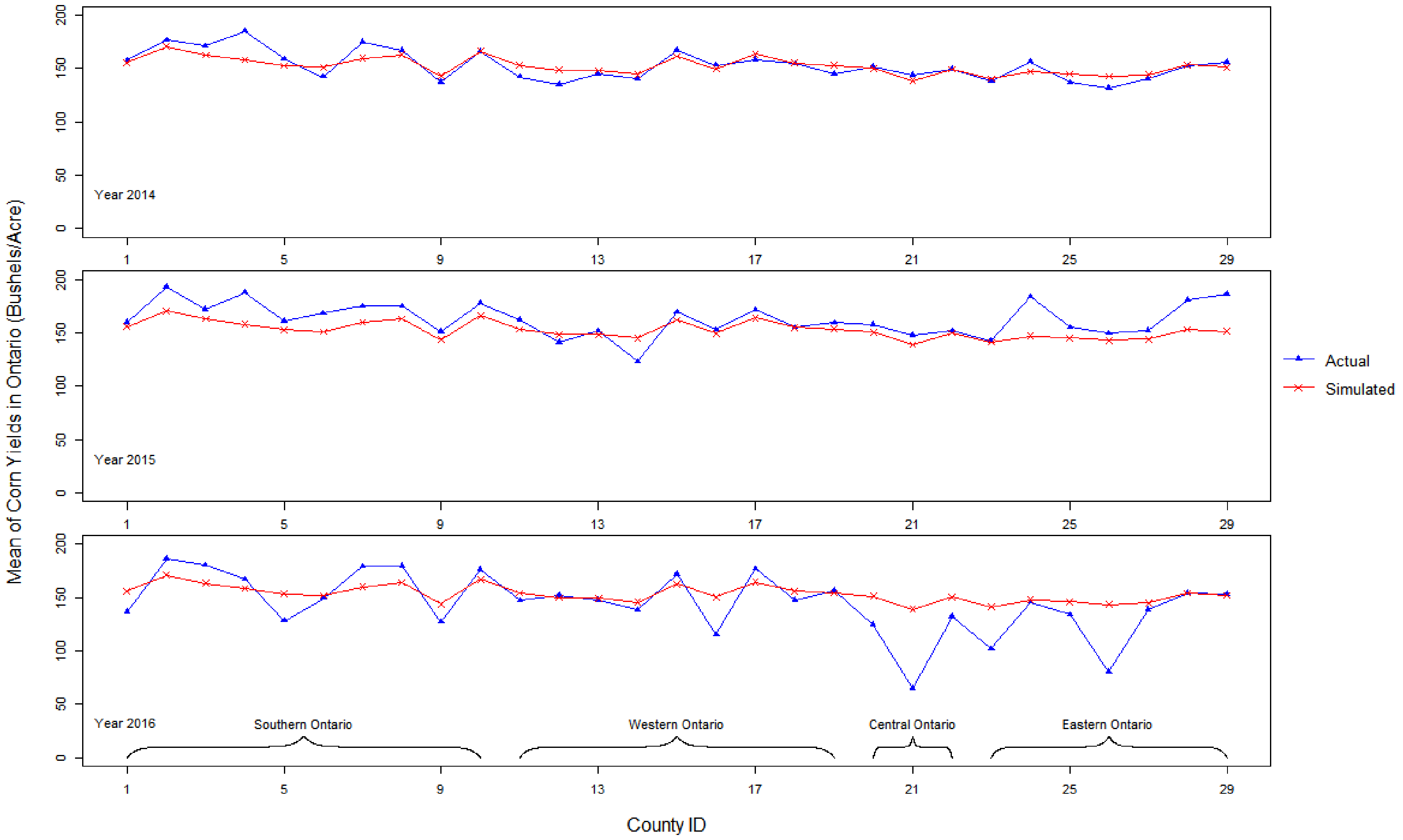

Source: Authors' calculation

Figure S3: County-Level Comparison Means of Actual Yields and Simulation Yields for Soybeans for 2014-2016

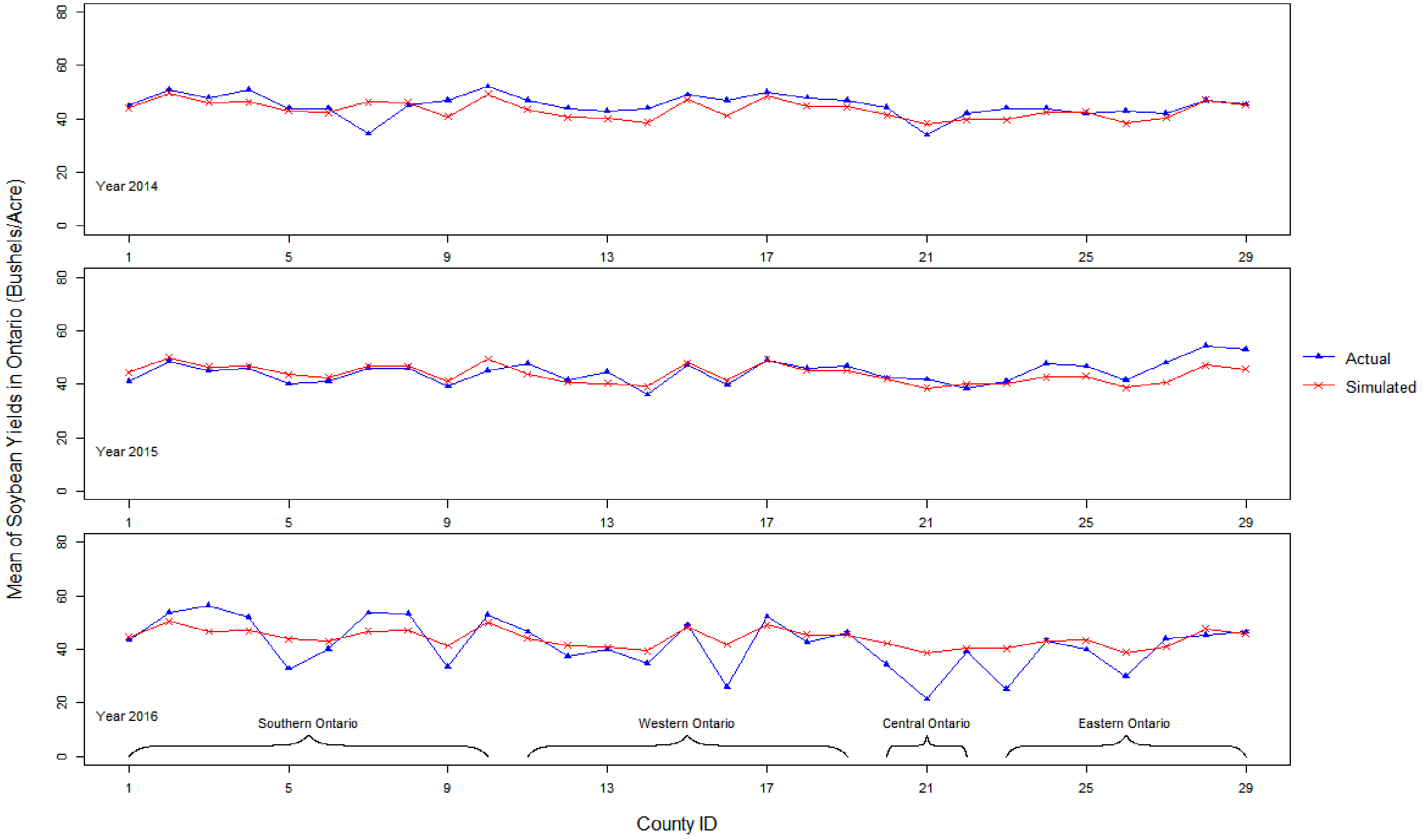

Source: Authors' calculation

Figure S4: The Boxplot of Average Deviation of Simulation Yields as Percentage of Actual Yields for Corn and Soybeans for 2014 – 2016 across 29 Counties

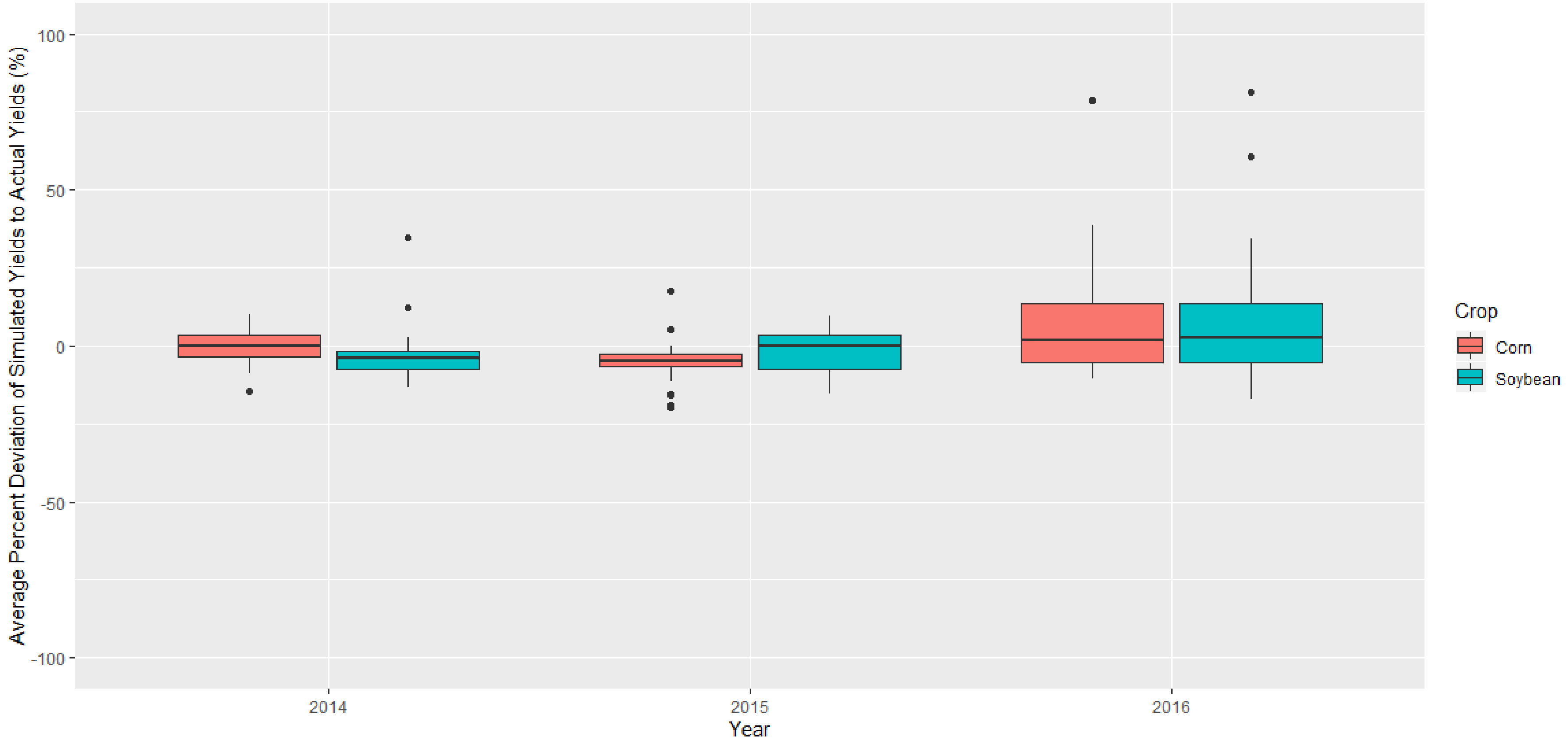

Source: Authors' calculation

Notes:

1. the averaged deviation of simulation yield is the difference between the means of actual yields and simulated yields for 500 simulation runs for one crop and one county. It is divided by the mean of actual yields and represents the deviation of simulation yield as a percentage of actual yields (i.e. (mean of simulated yields – mean of actual yields)/mean of actual yields * 100%).

Table S3: Comparison of Mean Squared Errors between Studied Period and Out-of-Sample Period for Corn and Soybeans

Mean Squared Error (MSE)	1950-2013 Regression Model	2014-2016 Out-of-Sample Simulation
**Corn**	163.68	294.17
**Soybeans**	16.88	25.01

Source: Authors' calculation

Table S4: Comparison of Normalized Root Mean Squared Errors among Alternative Time Periods for Corn and Soybeans

Mean Squared Error (MSE)	1959-2013 Regression Model	38 Years of 1959-2013 Training Data	17 Years of 1959-2013 Testing Data	2014-2016 Out-of-Sample Simulation
**Corn**	13.3%	13.6%	13.3%	11.2%
**Soybeans**	11.6%	11.7%	12.1%	11.4%

Source: Authors' calculation

Table S5: Correlation of Monthly CO_2 between Hawaii and Ontario for 2005-2016. The monthly CO_2 are highly correlated.

Jan	Feb	Mar	Apr	May	Jun	Jul	Aug	Sep	Oct	Nov	Dec
0.986	0.975	0.981	0.973	0.983	0.981	0.958	0.867	0.947	0.981	0.945	0.966

Table S6: Estimated Coefficients of Monthly CO_2 Concentration in Hawaii on the Monthly CO_2 Concentration in Egbert, Ontario with Monthly Dummies Base on May (N=139). The values in parentheses are standard errors. ***, **, and * indicate significant coefficients with significance level of 0.01, 0.05, and 0.1, respectively.

**Dependent Variable: Monthly CO_2 in Ontario**	
**Independent Variables**	**Estimated Coefficients**
Constant (May)	23.97**
	(10.05)
Monthly CO_2 in Hawaii	0.9413***
	(0.02549)
Monthly Dummies	
January	11.41***
	(0.9097)
February	9.329***
	(0.9067)
March	8.986***
	(0.8877)
April	6.712***
	(0.8864)
June	-8.479***
	(0.9047)
July	-15.37***
	(0.8865)
August	-14.14***
	(0.8908)
September	-4.066***
	(0.8953)
October	5.817***
	(0.8945)
November	12.25***
	(0.8935)
December	13.90***
	(0.9079)
Adjusted R-Squared	0.9688
F-Value	358.3
